# Reduced cortical complexity in patients with end-stage kidney disease prior to dialysis initiation

**DOI:** 10.3389/fnins.2022.971010

**Published:** 2022-10-31

**Authors:** Huijie Yuan, Haining Li, Junya Mu, Wen Gu, Xinyi Zhu, Lei Gao, Yuchen Zhang, Shaohui Ma

**Affiliations:** ^1^Department of Medical Imaging, First Affiliated Hospital of Xi’an Jiaotong University, Xi’an, China; ^2^Department of Radiology, Zhongnan Hospital of Wuhan University, Wuhan, China; ^3^Department of Nuclear Medicine, First Affiliated Hospital of Xi’an Jiaotong University, Xi’an, China

**Keywords:** magnetic resonance imaging, cortical complexity, fractal dimension, brain, memory impairment, end-stage kidney disease

## Abstract

End-stage kidney disease (ESKD) is associated with cognitive impairment (CI) and affects different aspects of cortical morphometry, but where these changes converge remains unclear. Fractal dimension (FD) is used to represent cortical complexity (CC), which describes the structural complexity of the cerebral cortex by integrating different cortical morphological measures. This study aimed to investigate changes in CC in patients with ESKD prior to initiation of dialysis and to evaluate the relationship between changes in CC, cognitive performance, and uremic toxins. Forty-nine patients with ESKD naive to dialysis and 31 healthy controls (HCs) were assessed using structural magnetic resonance imaging (MRI) and cognitive tests, including evaluations of global cognitive function, memory, and executive function. Clinical laboratory blood tests were performed on all patients with ESKD, including measurement of nine uremic toxin-related indices. CC was measured using MRI data to determine regional FD values. We estimated the association between cognitive performance, uremic toxin levels, and CC changes. Compared to HCs, patients with ESKD showed significantly lower CC in the left precuneus (*p* = 0.006), left middle temporal cortex (*p* = 0.010), and left isthmus cingulate cortex (*p* = 0.018). Furthermore, lower CC in the left precuneus was associated with impaired long-term delayed memory (Pearson *r* = 0.394, *p* = 0.042) in patients with ESKD. Our study suggests that regional decreases in CC are an additional characteristic of patients with ESKD naive to dialysis, related to impaired long-term memory performance. These findings may help further understand the underlying neurobiological mechanisms between brain structural changes and CI in patients with ESKD.

## Introduction

End-stage kidney disease (ESKD) is the most serious outcome of chronic kidney disease and requires renal replacement therapy (including dialysis and kidney transplantation). Patients with ESKD are at high risk of cognitive impairment (CI) (Viggiano et al., 2020). CI is associated with a high symptom burden, poor self-management, frequent hospitalizations, low quality of life, and high mortality in patients with ESKD (Kurella Tamura and Yaffe, 2011). The prevalence of CI, including mild cognitive impairment (MCI) and dementia, is approximately 10% in young patients (21–44 years) with ESKD (Kurella Tamura and Yaffe, 2011). Previous studies have suggested that CI in patients with ESKD was associated with vascular risk factors, uremic toxin-induced vascular injury, and neurotoxic effects (Bugnicourt et al., 2013; Lu et al., 2015). However, the neurobiological mechanisms of CI in patients with ESKD are still unclear.

Structural magnetic resonance imaging (MRI) can be used to quantitatively assess damage to brain morphometry to improve our understanding of the neurobiological mechanisms underlying CI in ESKD. Structural MRI studies of brain gray matter in patients with ESKD mainly focus on gray matter volume and cortical thickness. Various research methods were employed, such as voxel-based morphometry analysis and surface-based morphology analysis. Many studies have found that the results of injured brain regions in patients with ESKD are inconsistent, but they are mostly concentrated in the prefrontal cortex (Zhang et al., 2013; Qiu et al., 2014; Dong et al., 2018; Ding et al., 2020; Wang et al., 2022), temporal lobe (Qiu et al., 2014; Ding et al., 2020; Jin et al., 2020; Wang et al., 2022), limbic system (Ding et al., 2020; Wang et al., 2022), and subcortical structures such as insula (Zhang et al., 2013; Chai et al., 2015; Ding et al., 2020), thalamus (Jin et al., 2020; Gu et al., 2021), and basal ganglia (Chai et al., 2015; Richerson et al., 2021; Wang et al., 2022). Meanwhile, these studies also showed that cortical structural changes in patients with ESKD are associated with executive and memory dysfunction and uremic toxin accumulation, although the results differed.

Cortical complexity (CC) analysis is used to evaluate brain structure by describing surface complexity. It can be quantitatively measured using the fractal dimension (FD) of the surface of the cerebral cortex. The FD measure combines cortical features, such as cortical thickness, folded area, cortical gyrification, and sulcal depth, into an integrated index (Im et al., 2006). Based on the view that brain structure can be described mathematically as fractal (Kiselev et al., 2003), FD has the advantage of not relying on defining an explicit shell, thus avoiding confounding caused by the estimation of the regional gyrification index (Nicastro et al., 2020). Since the cerebral cortex is a convoluted surface in three-dimensional (3D) space, its FD is expected to lie between 2 and 3. Higher numerical values indicate higher levels of detail or irregularity of the cortical shape (Hedderich et al., 2020). Previous studies have shown a correlation between CC and cognitive performance. For example, a cohort study of volunteers without dementia found that higher FD values were associated with less cognitive decline (Mustafa et al., 2012). Decreased CC in later life is associated with information processing speed, auditory-language learning, etc. (Sandu et al., 2014). Furthermore, CC abnormality is considered an early fingerprint of neurodegeneration and has been observed in patients with neurological disorders, such as Alzheimer’s disease (King et al., 2009; Ruiz de Miras et al., 2017), MCI (Ruiz de Miras et al., 2017), frontotemporal dementia (Nicastro et al., 2020), and minimal hepatic encephalopathy (Chen et al., 2020). Brain injury in ESKD is affected by both vascular injury and neurodegeneration, which may lead to changes in CC. And altered CC may be associated with CI and uremic toxins. However, few studies have evaluated CC in patients with ESKD.

This study aimed to investigate changes in CC in patients with ESKD prior to initiation of dialysis and the relationship between CC changes, cognitive abilities, and uremic toxins levels in patients. Previous studies have mostly targeted dialysis patients. Patients with ESKD prior to initiation of dialysis were included in this study. We excluded confounding factors associated with dialysis to evaluate better the neurobiological mechanisms of brain injury associated with kidney-brain interactions. We hypothesized that patients with ESKD prior to initiation of dialysis would have CC changes compared to healthy controls (HCs), which is associated with certain CI and urea toxin accumulation.

## Materials and methods

### Participants

Each subject provided informed written consent prior to magnetic resonance scanning. Forty-nine patients with ESKD naive to dialysis were recruited from the Department of Nephrology, First Affiliated Hospital of Xi’an Jiaotong University. Thirty-one HCs matched for age, handedness (all right-handed), sex, and education level were recruited from the local community. All subjects participated in a cross-sectional study approved by our local ethics committee.

The inclusion criteria for patients with ESKD were: (1) estimated glomerular filtration rate < 15 ml/min/1.73 m^2^; (2) not yet on dialysis (including hemodialysis and peritoneal dialysis) or recipient of a kidney transplant; (3) aged 18–50 years. HCs did not have any renal system or other organ system diseases. The exclusion criteria were: (1) psychiatric or neurological diseases; (2) type I or type II diabetes; (3) history of alcohol addiction or drug abuse; (4) macroscopic brain T2-visible lesions on MRI scans; (5) contraindication of MRI examination; (6) unable to complete MRI examination and cognitive assessments.

### Blood samples collection and analysis

All patients with ESKD were evaluated using blood biochemical tests, including hemoglobin (g/L), hematocrit (%), and nine uremic toxin-related indices: blood urea nitrogen (BUN) (mmol/L), creatinine (μmol/L), uric acid (μmol/L), cystatin C (mg/L), calcium (mmol/L), phosphorus (mmol/L), potassium (mmol/L), sodium (mmol/L), and parathormone (pg/ml) within 24 h before the MRI.

### Neurocognitive assessments

All subjects completed a battery of standardized neurocognitive assessments administered by a neurologist with 10 years of experience before MRI scanning. Global cognitive function was measured using the Montreal cognitive assessment scale (MoCA) (Nasreddine et al., 2005). Memory was measured using the auditory verbal learning test-Huashan version (AVLT-H), which included immediate recall total score (IR-S, verbal working memory), short-term delayed recall score (SR-S, short-term delayed memory), long-term delayed recall score (LR-S, long-term delayed memory), and recognition score (REC-S, recognition). The AVLT-H was revised and developed based on the California verbal learning test to evaluate episodic memory (Guo et al., 2009). As a representative test for episodic memory, AVLT-H can detect memory deficits in normal aging and individuals with MCI and early Alzheimer’s disease (Guo et al., 2009; Zhao et al., 2015). The executive function was measured using the trail-making test, part A (TMT-A) (Bowie and Harvey, 2006).

### Image acquisition

All MRI datasets were scanned using a 3 Tesla GE Excite scanner (GE Medical Systems, Milwaukee, WI, USA) with an 8-channel head coil at the Department of Medical Imaging, First Affiliated Hospital of Xi’an Jiaotong University. All subjects underwent T2-weighted and T2-weighted fluid-attenuated inversion recovery imaging sequences to rule out brain lesions or abnormalities, then underwent 3D high-resolution T1-weighted scanning using a 3D fast spoiled gradient echo sequence with parameters as follows: 140 axial slices; repetition time = 10.8 ms; echo time = 4.8 ms; flip angle = 9°; slice thickness = 1.0 mm; no gap; matrix = 256 × 256; and field of view = 256 mm × 256 mm.

### Image processing

All original 3D high-resolution T1-weighted images saved as DICOM files were converted to Nifti-format using dcm2nii (Li et al., 2016). The reoriented images were then processed and analyzed using the Computational Anatomy Toolbox 12 (version CAT12.7, r1739,^1^) implemented in Statistical Parametric Mapping software (SPM12,^2^) for Matlab (version 2018a, the MathWorks Inc., Natick, USA) with processing pipeline for surface-based morphometry. These images underwent tissue segmentation into gray matter, white matter and cerebrospinal fluid, and the spherical harmonic method was used for topological correction (Yotter et al., 2011). Diffeomorphic anatomical registration through exponentiated lie algebra (DARTEL) algorithm was then used to the surface for spherical registration (Ashburner, 2007). After that, spherical harmonic reconstruction was used to measure the regional FD (Yotter et al., 2011). All reconstructed FD surfaces were smoothed with a Gaussian kernel of 20 mm full width at half maximum. All participants passed both the visual and the CAT12 data quality checks. The mean FD value within each of the 68 regions of interest (ROI) defined by the Desikan-Killiany Atlas (Desikan et al., 2006) was extracted.

### Statistical analysis

SPSS software (version 26.0; IBM Corp., Armonk, NY, USA) was used for statistical analysis. The Shapiro–Wilk test was used to evaluate normality, and Levene’s test was used to determine the equality of variances. Group differences for quantitative variables conforming to normal distribution were determined using independent two-sample *t*-tests, while data were not conforming to a normal distribution using the Mann–Whitney *U* test. Group differences for categorical variables were assessed using the Chi-square test. Group differences for neurocognitive data were then evaluated using a one-way analysis of covariance (ANCOVA) adjusted for sex, age, and education level. *P* < 0.05 was considered statistically significant.

Group differences for CC values (ROI-based analysis) were evaluated using ANCOVA adjusted for sex, age, and education level. Results corrected for multiple comparisons using the false discovery rate (FDR). We set the FDR corrected *p*-value < 0.05 as the statistical threshold.

The correlations among changes in CC, cognition assessments (IR-S, SR-S, LR-S, REC-S, TMT-A, and MoCA), and uremic toxin levels (BUN, creatinine, uric acid, cystatin C, calcium, phosphorus, potassium, sodium, and parathormone) in the patients with ESKD were explored using Pearson correlation analysis for normally distributed data or Spearman correlation analysis for non-normally distributed data. Partial Pearson correlation was used to explore the relationship between the results of cognition assessments and uremic toxin levels and changes in CC in patients with ESKD adjusted for sex, age, and education level. Partial Spearman correlation was used for non-normally distributed data. FDR-corrected *p*-value < 0.05 was considered statistically significant.

## Results

### Demographic, clinical characteristics and cognitive assessments

**FIGURE 1:**
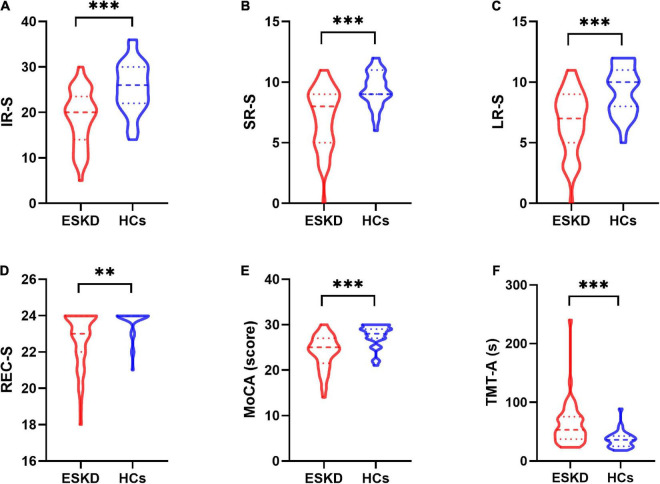
The violin plot depicts group differences in neurocognitive tests between patients with ESKD and HCs after adjusting for age, sex, and education level. **(A)** The ESKD group had lower IR-S than HCs (*p* < 0.001). **(B)** The ESKD group had lower SR-S than HCs (*p* < 0.001). **(C)** The ESKD group had lower LR-S than HCs (*p* < 0.001). **(D)** The ESKD group had lower REC-S than HCs (*p* = 0.006). **(E)** The ESKD group had a lower MoCA score than HCs (*p* < 0.001). **(F)** The ESKD group had longer TMT-A than HCs (*p* < 0.001). ESKD, end-stage kidney disease; HCs, healthy controls; IR-S, immediate recall total score; SR-S, short-term delayed recall score; LR-S, long-term delayed recall score; REC-S, recognition score; MoCA, Montreal cognitive assessment; TMT-A, trail-making test, part A. **p < 0.01; ***p < 0.001.

Group differences in demographic information and clinical characteristics are summarized in Table 1. There were no significant differences in age (*p* = 0.702), sex (*p* = 0.618), education level (*p* = 0.195), or body mass index (BMI) (*p* = 0.621) between the ESKD and HCs groups. Group differences in neurocognitive tests are shown in Table 2. The ESKD group showed poorer memory performance in IR-S (*p* < 0.001), SR-S (*p* < 0.001), LR-S (*p* < 0.001), and REC-S (*p* = 0.006) after adjusting for age, sex, and education level comparing to HCs group (Figures 1A–D). In addition, the ESKD group showed worse global cognitive function and executive function than the HCs group (MoCA: *p* < 0.001; TMT-A: *p* < 0.001; Figures 1E,F) after adjusting for age, sex, and education level.

**TABLE 1:** Demographic information and clinical characteristics in patients with ESKD and HCs.

Variables	ESKD (*n* = 49)	HCs (*n* = 31)	*p*-value
Sex (men/women)	29/20	17/14	0.702^a^
Age (year, mean ± SD)	37.69 ± 10.53	36.52 ± 9.78	0.618^b^
Education (year)	10.88 ± 3.61	12.39 ± 4.70	0.195^c^
BMI (kg/m^2^)	23.02 ± 3.26	23.52 ± 6.04	0.621^c^
Duration (months)	72.12 ± 118.74	–	–
Hemoglobin (g/L)	90.39 ± 17.76	–	–
Hematocrit (%)	27.03 ± 5.69	–	–
BUN (mmol/L)	29.05 ± 9.32	–	–
Creatinine (μmol/L)	795.98 ± 293.27	–	–
Uric acid (μmol/L)	491.90 ± 150.12	–	–
Cystatin C (mg/L)	4.07 ± 0.96	–	–
Calcium (mmol/L)	1.99 ± 0.27	–	–
Phosphorus (mmol/L)	1.78 ± 0.51	–	–
Potassium (mmol/L)	4.65 ± 0.72	–	–
Sodium (mmol/L)	140.10 ± 3.88	–	–
Parathormone (pg/ml)	306.17 ± 217.92	–	–

ESKD, end-stage kidney disease; HCs, healthy controls; BMI, body mass index; BUN, blood urea nitrogen. All quantitative data were expressed as the mean ± standard deviation; numbers for sex data. ^a^Analyzed using the Chi-square test. ^b^ Analyzed using the independent two-sample *t*-tests. ^c^Analyzed using the Mann–Whitney *U* test.

**TABLE 2:** Cognitive assessments in patients with ESKD and HCs.

Variables	ESKD (*n* = 49)	HCs (*n* = 31)	Unadjusted *p*-value	*F*-value	Adjusted *p*-value
**AVLT-H**					
IR-S	18.80 ± 6.05 (17.76, 20.58)	25.45 ± 5.43 (23.08, 26.64)	< 0.001	24.365	< 0.001
SR-S	7.04 ± 2.50 (6.57, 7.70)	9.42 ± 1.46 (8.56, 9.98)	< 0.001	21.751	< 0.001
LR-S	6.71 ± 2.55 (6.22, 7.41)	9.23 ± 1.98 (8.31, 9.82)	< 0.001	21.469	< 0.001
REC-S	22.73 ± 1.52 (22.41, 23.41)	23.68 ± 0.75 (23.15, 24.07)	< 0.001	7.874	0.006
MoCA	23.92 ± 3.72 (23.32, 24.87)	27.65 ± 2.48 (26.38, 28.35)	< 0.001	26.556	< 0.001
TMT-A (s)	62.24 ± 36.17 (53.25, 67.94)	36.59 ± 14.39 (29.91, 48.47)	< 0.001	12.746	< 0.001

ESKD, end-stage kidney disease; HCs, healthy controls; AVLT-H, auditory verbal learning test-Huashan version; IR-S, immediate recall total score; SR-S, short-term delayed recall score; LR-S, long-term delayed recall score; REC-S, recognition score; MoCA, Montreal cognitive assessment; TMT-A, trail-making test, part A. All quantitative data were expressed as the mean ± standard deviation. Unadjusted *p*-values were obtained using the two-sample *t*-tests or the Mann–Whitney *U* test. Adjusted *p*-values were calculated using a one-way analysis of covariance (ANCOVA) adjusted for sex, age, and education level. Data in parentheses were 95% confidence intervals.

### Group differences in cortical complexity

**FIGURE 2:**
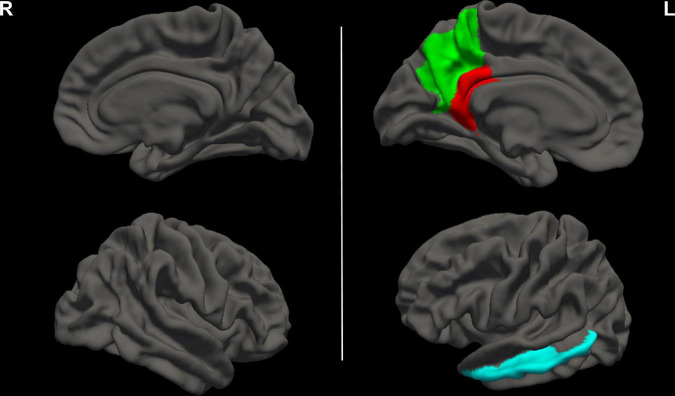
Brain regions with decreased CC values in patients with ESKD compared to HCs. These results were corrected for multiple comparisons (FDR-corrected *p* < 0.05). The green area represented the left precuneus. The blue area represented the left middle temporal cortex. The red area represented the left isthmus cingulate cortex. R, right; L, left; CC, cortical complexity; ESKD, end-stage kidney disease; HCs, healthy controls; FDR, false discovery rate.

**FIGURE 3:**
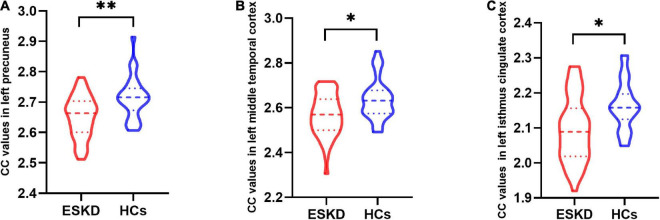
The violin plot depicts group differences in cortical complexity (CC) values in brain regions between patients with end-stage kidney disease (ESKD) and healthy controls (HCs) after adjusting for age, sex, and education level. **(A)** The ESKD group had lower CC values in the left precuneus compared to HCs (2.65 ± 0.07 vs. 2.71 ± 0.07, false discovery rate-corrected *p* = 0.006). **(B)** The ESKD group had lower CC values in the left middle temporal cortex compared to HCs (2.57 ± 0.09 vs. 2.64 ± 0.09, FDR-corrected *p* = 0.010). **(C)** The ESKD group had lower CC values in the left isthmus cingulate cortex compared to HCs (2.09 ± 0.09 vs. 2.16 ± 0.07, FDR-corrected *p* = 0.018). *p < 0.05 and **p < 0.01. CC, cortical complexity; ESKD, end-stage kidney disease; HCs, healthy controls; FDR, false discovery rate.

Group differences in CC values are shown in Table 3 and Figure 2. Compared with HCs, CC values were significantly lower in the left precuneus (2.65 ± 0.07 vs. 2.71 ± 0.07, FDR-corrected *p* = 0.006, Figure 3A), left middle temporal cortex (2.57 ± 0.09 vs. 2.64 ± 0.09, FDR-corrected *p* = 0.010, Figure 3B), and left isthmus cingulate cortex (2.09 ± 0.09 vs. 2.16 ± 0.07, FDR-corrected *p* = 0.018, Figure 3C) in patients with ESKD after adjusting for age, sex, and education level. No regions showed significantly increased CC in patients with ESKD compared to HCs.

**TABLE 3:** Brain regions with significant differences in CC values between patients with ESKD and HCs.

CC values	Anatomic regions	ESKD (*n* = 49)	HCs (*n* = 31)	Side	Brodmann area	*F*-value	Adjust *p*-value	FDR-corrected *p*-value
ESKD vs. HCs (FDR correction with 68 iterations, *p* < 0.05)								
ESKD < HCs	Precuneus	2.65 ± 0.07	2.71 ± 0.07	L	7	15.634	< 0.001	0.006
	Middle temporal cortex	2.57 ± 0.09	2.64 ± 0.09	L	21	15.971	< 0.001	0.010
	Isthmus cingulate gyrus	2.09 ± 0.09	2.16 ± 0.07	L	26,29,30	12.259	< 0.001	0.018

CC, cortical complexity; ESKD, end-stage kidney disease; HCs, healthy controls; FDR, false discovery rate; L, left. All quantitative data were expressed as the mean ± standard deviation. Adjusted *p*-values were calculated using a one-way analysis of covariance (ANCOVA) adjusted for sex, age, and education level.

### Correlations between decreased cortical complexity and cognitive performance and uremic toxin levels

**FIGURE 4:**
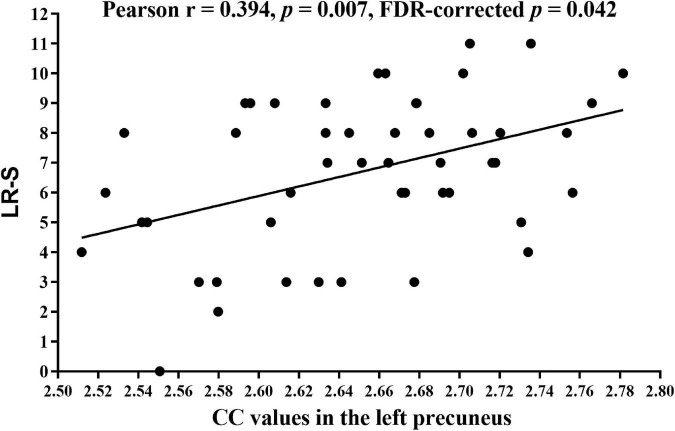
Correlation analysis between the CC values of the left precuneus and LR-S in patients with ESKD. Pearson correlation analysis showed lower CC values of the left precuneus were associated with lower LR-S (Pearson *r* = 0.394, *p* = 0.007, FDR-corrected *p* = 0.042) after adjusting for age, sex, and education level. CC, cortical complexity; LR-S, long-term delayed recall score; ESKD, end-stage kidney disease; FDR, false discovery rate.

There was a significant correlation between CC values in the left precuneus and LR-S in patients with ESKD (Pearson *r* = 0.394, *p* = 0.007, FDR-corrected *p* = 0.042, Figure 4) after adjusting for age, sex, and education level. There were no significant correlations between CC in the left precuneus and other measures of cognitive performance and uremic toxin levels. Other brain regions with significant CC changes were not statistically significantly associated with cognitive tests and uremic toxin levels.

## Discussion

We aimed to explore changes in CC in patients with ESKD naive to dialysis, and the relationship between CC changes, cognitive abilities, and uremic toxins levels in patients. Consistent with our hypothesis, patients with ESKD showed significant CC reductions compared to HCs in several brain regions, including the left precuneus, the left middle temporal cortex, and the left isthmus cingulate cortex. Furthermore, decreased CC values in the left precuneus were associated with impaired long-term memory function in patients with ESKD naive to dialysis. Our study suggested that regional changes in CC are an additional feature of patients with ESKD prior to initiation of dialysis. Abnormal local CC may be the neurobiological basis of CI in patients with ESKD.

Previous studies on brain structure in patients with ESKD have found diffusely decreased gray matter volume and cortical thickness compared to HCs. These results included abnormal brain regions in our study. Our findings further suggested that patients with ESKD have cortical morphological changes in multiple brain regions regarding CC. It is well-known that brain injury in patients with ESKD is due to a combination of cerebrovascular injury and neurodegenerative changes associated with Alzheimer’s disease (Bugnicourt et al., 2013; An et al., 2017). The pathological processes included endothelial dysfunction and direct toxic effects of uremic toxins (Bugnicourt et al., 2013). Therefore, one explanation of these changes is that a combination of factors such as neuronal cell injury, brain amyloid deposition, oxidative stress, inflammatory response, and toxin effects may result in abnormal processes of surface geometry. Different MRI techniques provide supporting evidence for this inference. For example, a significant reduction of the NAA/Cr ratio in the gray matter was indicative of neuronal cell injury or neurodegeneration in patients with ESKD using proton magnetic resonance spectroscopy (Geissler et al., 1995). Additionally, functional MRI studies have shown that brain injury in patients with ESKD has some commonalities in structure and function. For instance, Ni et al. (2014) found abnormal internal functional connectivity in the default mode network consisting of brain regions such as the posterior cingulate cortex, precuneus and prefrontal cortex in patients with ESKD. CC integrates many cortical features and represents changes in cortical geometry in the lifespan (Mustafa et al., 2012). Studies have shown that gray matter CC in the left hemisphere increases until adolescence. In contrast, gray matter CC in the right hemisphere increases until the middle of the third decade of life, and both then decrease with aging and many diseases can exacerbate the decrease in CC (Ziukelis et al., 2022). The ESKD groups included in this study were age-matched to HCs, and we explored CC differences at the group level based on these two populations. Additionally, our age span is a stage of young adults, and this age difference may have little impact on CC. Furthermore, this age difference is likely significantly smaller than major neuropsychiatric or systemic diseases, such as ESKD, in this study. We speculated that this decrease in CC would be more severe, and the extent of the injury would be greater as patients with ESKD become older. Further studies are needed to explore the effect of age on CC in patients with ESKD.

We found that reduced CC of the left precuneus positively correlated with impaired long-term memory in patients with ESKD naive to dialysis. That is different from previous research results. Zhang et al. (2013) found diffusely abnormal gray matter volume was associated with attention dysfunction. Ding et al. (2020) found cortex thickness in the right inferior parietal lobular is positively correlated with immediate recall memory (IR-S). It may be related to differences in sample selection and methods. But these results are interpretable. As a key part of the default mode network (Buckner et al., 2008), the precuneus plays an important role in a diverse array of highly integrated functions, including episodic memory retrieval, visuospatial imagery, self-processing, and consciousness (Cavanna and Trimble, 2006; Hebscher et al., 2020). A previous study (O’Lone et al., 2016) has indicated that compared to HCs, patients with ESKD show pronounced deficits in some cognitive domains, especially explicit memory (recalling a list of words). These were consistent with our findings. Storage and retrieval of explicit memories, including episodic memory, require the proper function of the cerebral cortex and hippocampus and appropriate activity of cholinergic neurons in the basal nucleus of Meynert (Viggiano et al., 2020). It may explain why our study found an association between decreased CC in the left precuneus and impaired long-term episodic memory impairment. Additionally, previous studies found brain structural abnormalities associated with uremic toxins levels, including serum urea (Zhang et al., 2013; Dong et al., 2018) and serum creatinine (Dong et al., 2018). Unfortunately, we did not find significant correlations between CC changes and uremic toxin levels. It may be due to the small sample size of this study.

Our study was subject to several limitations. First, this study was an observational study with a small sample size. Future studies should include larger sample sizes and longitudinal comparative studies that include groups before and after dialysis. It would allow for a better evaluation of the relationship between brain structural abnormalities and cognitive changes before and after dialysis in patients with ESKD. Second, the patients with ESKD included in this study were younger. The risk of CI increases with age, and structural brain abnormalities may become more severe. Therefore, future studies should include the elderly population to explore further the relationship between brain structural abnormalities, uremic toxins, and CI. Third, this study did not stratify the severity of CI. More studies are needed to characterize the associations between the degree of structural abnormality and the progression of CI. Finally, there are few studies on whether CC recovers in patients with ESKD after dialysis or kidney transplantation. Studying this question may help further explore the potential neurobiological mechanism of CI in patients with ESKD.

## Conclusion

This study demonstrated that regional decreases in CC are an additional characteristic of patients with ESKD naive to dialysis, related to impaired long-term memory function. These findings may help further understand the underlying neurobiological mechanisms between brain structural changes and CI in patients with ESKD. Therefore, we suggested that measuring CC may serve as an additional indicator to explore CI in ESKD.

## Data availability statement

The raw data supporting the conclusions of this article will be made available by the authors, without undue reservation.

## Ethics statement

The studies involving human participants were reviewed and approved by the Medical Ethics Committee of the First Affiliated Hospital of the Medical College in Xi’an Jiaotong University. The patients/participants provided their written informed consent to participate in this study.

## Author contributions

HY, HL, YZ, and SM designed the study. HY and HL performed the data analysis and drafted the manuscript. HY, HL, and JM participated in data analysis. WG, XZ, LG, YZ, and SM critically revised the manuscript. All authors reviewed the final manuscript.

## Footnotes

1
https://neuro-jena.github.io/cat/

2
https://www.fil.ion.ucl.ac.uk/spm/

## Abbreviations

ESKD: end-stage kidney disease
CC: cortical complexity
FD: fractal dimension
CI: cognitive impairment
MCI: mild cognitive impairment
MRI: magnetic resonance imaging
HCs: healthy controls
BUN: blood urea nitrogen
3D: three-dimensional
MoCA: Montreal cognitive assessment scale
AVLT-H: auditory verbal learning test-Huashan version
IR-S: immediate recall total score
SR-S: short-term delayed recall score
LR-S: long-term delayed recall score
REC-S: recognition score
TMT-A: trail-making test, part A
ROI: regions of interest
ANCOVA: one-way analysis of covariance
FDR: false discovery rate.
